# Prevalence, Patterns, and Predictors of SARS-CoV-2 RNA and Culturable Virus in Tears of a Case-Ascertained Household Cohort

**DOI:** 10.1016/j.ajo.2024.04.008

**Published:** 2024-04-23

**Authors:** MATTHEW SO, SARAH A. GOLDBERG, SCOTT LU, MIGUEL GARCIA-KNIGHT, MICHELLE C. DAVIDSON, MICHEL TASSETTO, VICTORIA WONG MURRAY, KHAMAL ANGLIN, JESUS PINEDA-RAMIREZ, JESSICA Y. CHEN, PAULINA R. RUGART, EUGENE T. RICHARDSON, MELISSA BRIGGS-HAGEN, CLAIRE M. MIDGLEY, RAUL ANDINO, GERAMI D. SEITZMAN, JOHN GONZALES, MICHAEL J. PELUSO, JEFFREY N. MARTIN, JOHN DANIEL KELLY

**Affiliations:** Institute for Global Health Sciences, University of California, San Francisco, California, USA; Institute for Global Health Sciences, University of California, San Francisco, California, USA; Department of Epidemiology and Biostatistics, University of California, San Francisco, San Francisco, California, USA; Institute for Global Health Sciences, University of California, San Francisco, California, USA; Department of Epidemiology and Biostatistics, University of California, San Francisco, San Francisco, California, USA; Department of Microbiology and Immunology, University of California, San Francisco, San Francisco, California, USA; School of Medicine, University of California, San Francisco, San Francisco, California, USA; Department of Microbiology and Immunology, University of California, San Francisco, San Francisco, California, USA; School of Medicine, Cooper Medical School of Rowan University, Camden, New Jersey, USA; Institute for Global Health Sciences, University of California, San Francisco, California, USA; Institute for Global Health Sciences, University of California, San Francisco, California, USA; Institute for Global Health Sciences, University of California, San Francisco, California, USA; Department of Epidemiology and Biostatistics, University of California, San Francisco, San Francisco, California, USA; Department of Global Health and Social Medicine, Harvard Medical School, Boston, Massachusetts, USA; Division of Infectious Diseases, Brigham and Women’s Hospital, Boston, Massachusetts, USA; Coronavirus and Other Respiratory Viruses Division, National Center for Immunization and Respiratory Diseases, Centers for Disease Control and Prevention, Atlanta, Georgia, USA; Coronavirus and Other Respiratory Viruses Division, National Center for Immunization and Respiratory Diseases, Centers for Disease Control and Prevention, Atlanta, Georgia, USA; Department of Microbiology and Immunology, University of California, San Francisco, San Francisco, California, USA; F.I. Proctor Foundation, University of California, San Francisco, San Francisco, California, USA; Department of Ophthalmology, University of California, San Francisco, California, USA; F.I. Proctor Foundation, University of California, San Francisco, San Francisco, California, USA; Department of Ophthalmology, University of California, San Francisco, California, USA; Division of HIV, Infectious Diseases and Global Medicine, Zuckerberg San Francisco General Hospital, San Francisco, California, USA; Department of Epidemiology and Biostatistics, University of California, San Francisco, San Francisco, California, USA; Institute for Global Health Sciences, University of California, San Francisco, California, USA; Department of Epidemiology and Biostatistics, University of California, San Francisco, San Francisco, California, USA; F.I. Proctor Foundation, University of California, San Francisco, San Francisco, California, USA; Division of Hospital Medicine, San Francisco Veterans Affairs Medical Center, San Francisco, California, USA

## Abstract

**PURPOSE:**

To investigate the prevalence, patterns, and predictors of SARS-CoV-2 RNA and culturable virus in tears of a case-ascertained household cohort.

**DESIGN:**

Prospective, longitudinal case-ascertained household cohort identified through convenience sampling.

**METHODS:**

This analysis was restricted to individuals who were non-hospitalized, symptomatic, and tested positive for SARS-CoV-2 by nasal RT-PCR. Tears and anterior nasal biospecimens were serially collected throughout the acute period. Tears specimens were collected by the study staff using Schirmer test strips, and nasal specimens were self-collected. For both, SARS-CoV-2 RNA was quantified using qRT-PCR, and culturable virus was detected using presence of cytopathic effect (CPE) in tissue culture; positive CPE was confirmed by a qRT-PCR step. A series of cross-sectional unadjusted analyses were performed investigating the relationship between different sociodemographic determinants and biological factors associated with tears RNA positivity.

**RESULTS:**

Among the 83 SARS-CoV-2 infected participants, 10 (12%) had at least one RNA-positive tears specimen. Amongst these 10, 5 (50%) had concurrent presence of culturable virus, at a median of 7 days postsymptom onset (IQR: 4-7 days) (absolute range: 4-8 days).

**CONCLUSIONS:**

In this longitudinal cohort, we found evidence of culturable virus in the tears of a small proportion of nonhospitalized SARS-CoV-2 infected individuals. Current public health infection precautions do not account for transmission via tears, so these findings may improve our understanding of potential sources of SARS-CoV-2 transmission and contribute to developing future guidelines.

Although SARS-CoV-2 primarily spreads through respiratory droplets,^1^ tears and mucous membranes are other potential infectious sources.^2,3^ The few studies which have assessed SARS-CoV-2 presence in tears concluded that tears are likely insignificant contributors to transmission, but these studies were largely performed in the inpatient setting and rarely assessed infectiousness. These studies found that SARS-CoV-2 RNA could be recovered from the tears in less than 10% of hospitalized patients.^4,5^ In addition, there are conflicting findings about the presence of ocular disease involvement when SARS-CoV-2 RNA is also present.^4,5^ Thus, there is insufficient evidence regarding the prevalence, patterns and predictors of SARS-CoV-2 RNA and culturable virus in tears, particularly in the non-hospitalized population.

The objective of this analysis was to study the detection of SARS-CoV-2 RNA and culturable virus in tears of non-hospitalized participants, serially collected during the acute infectious period, from those participating in a longitudinal cohort study of acute SARS-CoV-2 infection. An additional objective of this analysis was to explore patient factors associated with detection of RNA or culturable virus in tears.

## METHODS

### ETHICS:

The study was reviewed by the University of California, San Francisco (UCSF) Institutional Review Board and given a designation of public health surveillance according to federal regulations as summarized in 45 Code of Federal Regulations 46.102(d)(1)(2). This activity was reviewed by CDC and was conducted consistent with applicable federal law and CDC policy.^6^ Written informed consent was obtained from all participants (IRB#20-30388, prospectively obtained from UCSF IRB).

### STUDY DESIGN:

This study used data from an observational, longitudinal cohort study that follows the natural history of acute SARS-CoV-2 infection in San Francisco. A case-ascertained household cohort was identified through convenience sampling and formed the basis of the study design. Detailed study procedures have been published elsewhere.^7,8^

### PROCEDURES:

Starting in September 2020, individuals of all ages who tested positive for SARS-CoV-2 were identified via molecular testing at UCSF-affiliated testing sites. Individuals were considered index cases if they were non-hospitalized, resided with at least one household member, and had no household members with COVID-19 symptoms in the prior week. Index cases and household contacts were eligible if enrolled within five days of symptom onset. This analysis was restricted to individuals of all ages who were symptomatic and tested positive for SARS-CoV-2 by nasal RT-PCR. Individuals who asked to be excluded from the study and from whom we were unable to collect tears specimens were also excluded from this analysis cohort (Supplementary Figure 1).

### MEASUREMENTS:

Tears and anterior nasal biospecimens were collected from participants using standardized methods. Tears were collected by study staff at home visits on day (d) of enrollment (dE), d9, d14, d21, d28. These visits were timed relative to the index case’s symptom onset. Tears specimens were collected using a Schirmer paper test strips,^9^ where filter paper was placed upon the inferolateral lid margins of both eyes for 5 minutes without anesthetic. Strips were then placed into viral transport medium (VTM) and stored at −80 °C. Tears samples from both eyes were pooled together for analyses.

Study staff trained participants to self-collect anterior nasal specimens daily from dE through d14, and on d17, d19, d21, and d28. The details of nasal specimen storage and collection have been previously reported.^7^ Briefly, after self-collection, samples were stored in study-dedicated freezers at −20 °C, after the study visit, they were transported on dry ice and stored at−80 °C.

Using previously reported methods to analyze both nasopharyngeal and tears specimens,^7^ we quantified SARS-CoV-2 RNA using reverse-transcription polymerase chain reaction (qRT-PCR) targeting the nucleocapsid (N) and envelope (E) genes. We characterized culturable virus as a qualitative value (yes/no culturable virus) using the presence of cytopathic effect (CPE) in the tissue culture; positive CPE results were confirmed as SARS-CoV-2 by an additional qRT-PCR step. More details are published elsewhere.^7^

### STATISTICAL ANALYSES:

We described the sociodemographic characteristics of the cohort, stratified by those with and without tears RNA positivity. If at least one of the tears specimens was positive for SARS-CoV-2 RNA by PCR, we classified the participant as “ever RNA positive in tears.” We performed a detailed epidemiological, clinical, and biological characterization of those who were ever RNA positive in tears, including time since symptom onset to tears collection, presence of medical comorbidities, and viral RNA load and CPE presence in the tears and anterior nasal biospecimens.

A series of cross-sectional unadjusted analyses were performed that investigated the relationship between different sociodemographic determinants (possible predictors) and tears RNA positivity. Each model included one of the sociodemographic variables and had an outcome of ever tears RNA positivity. Generalized linear models (GLM) with a modified Poisson (log link) were used to generate prevalence ratios (Supplementary Table 2).

A pooled cross-sectional adjusted analysis using modified Poisson GLMs, that clustered on participant and adjusted for both the age of participant and days post symptom onset (PSO), were used to investigate the relationship between concurrent (average +/− 1 day) nucleocapsid (N) nasal viral load and tears RNA positivity (Supplementary Table 2). All analyses were performed using STATA/SE 18.0 (StataCorp).

## RESULTS

The analysis cohort included 81 participants with SARS-CoV-2 infection enrolled from September 2020 to July 2021 (Supplementary Figure 1). The median age of the cohort was 38 years. Forty-three individuals (53%) were female, and 38 (47%) were non-Hispanic White. Among the individuals with SARS-CoV-2 infection, 29 (36%) had received their full initial series of SARS-CoV-2 vaccination prior to infection (defined as 2 doses of an mRNA vaccine or 1 dose of a viral vector vaccine). Participants were infected with Pre-Delta (N = 54, 67%) and Delta lineages (N = 27, 33%) (Supplementary Table 1).

### PREVALENCE AND PATTERNS OF SARS-COV-2 POSITIVITY IN TEARS:

Among the 81 SARS-CoV-2 infected participants, ten (12%) had RNA positivity on PCR testing in at least one of their tears specimens; only a single individual had tears positivity at more than one timepoint (d7 and d13). Three (30%) of these individuals were infected with a Delta lineage, 4 (40%) had received their full initial series of COVID-19 vaccination prior to SARS-CoV-2 infection, and 5 (50%) had the presence of at least one medical comorbidity (Supplementary Table 2).

Among the 10 participants with SARS-CoV-2 RNA positivity in tears, the median tears N-gene viral load was 3.43 log copies/mL (IQR: 3.15-4.49 log copies/mL) (absolute range: 1.98-6.69 log copies/mL); the median timing of RNA-positive tears sample was 7 days postsymptom onset (IQR: 4-13 days) (absolute range: 1-18 days); the median cycle-threshold value (Ct) was 34.14 (IQR: 32.65-35.18) (absolute range: 24.21-38.11). While in most cases, those with tears positivity were also positive from nasopharyngeal swab, in 3 cases we identified tears positivity in the absence of concurrent SARS-CoV-2 RNA nasal positivity. The median nasal N-gene viral RNA load was 6.02 log copies/mL (IQR: 4.59-7.81 log copies/mL) (absolute range: 3.72-7.99 log copies/mL). N-gene and E-gene viral load were similar both in the tears and nasal samples. Generally, amongst individuals who were both tears and nasal PCR positive, their nasal viral RNA load values were higher compared to their tears viral load (Table).

Among the 10 participants with RNA positivity in tears, five (50%) had the presence of culturable virus (CPE positive) identified in their tears-positive sample, and all five of these individuals had the presence of culturable virus identified in their corresponding nasal sample (Table). Of the five infectious participants, two (40%) were infected with a Delta lineage, two had received their full initial series of COVID-19 vaccinations prior to enrollment, and three had the presence of a comorbidity. None had more than one CPE-positive sample. The median of CPE-positive samples was also 7 days post symptom onset (IQR: 4-7 days) (absolute range: 4-8 days). Additionally, CPE-positive tears samples had higher viral RNA load in the tears, compared to CPE negative samples (median 4.49 vs 3.28 log copies/mL; *P*-value = .08) (Table).

### PREDICTORS OF SARS-COV-2 RNA POSITIVITY IN TEARS:

In cross-sectional analyses investigating sociodemographic and biological factors, only two determinants (cancer and kidney disease) were potentially associated with RNA-positive in tears. Individuals who reported cancer for which they received treatment in the 2 years prior to their COVID-19 diagnosis were approximately 5 times as likely to have a positive tears specimen compared to those without cancer (2/71 (3%) vs 2/10 (20%)). A similar result was observed for pre-existing kidney disease (1/71 (1%) vs 1/10 (10%)) (Supplementary Table 2).

In pooled cross-sectional analyses investigating the relationship between nasal viral load and RNA-positivity in tears, no statistically significant association was found (Supplementary Table 2).

## DISCUSSION

In this study, we identified SARS-CoV-2 RNA in the tears of 10 outpatients with COVID-19 studied through the Delta wave of the pandemic. We found that tears could harbor infectious virus in a small subset. Although the prevalence of SARS-CoV-2 in tears was low (12%), the proportion with culturable virus as determined by CPE was high (50%). Additionally, we found evidence of prolonged presence of culturable virus in the tears among nonhospitalized SARS-CoV-2 infected individuals.^10^

The prevalence of SARS-CoV-2 tears RNA positivity in our cohort is similar to previous reports among individuals hospitalized with COVID-19 (approximately 10%).^4,5^ As compared to the median Ct values in our study (34.14), Ct values from other studies in inpatient studies have been shown to be similar (33-34).^11^ Although there are limitations to comparing Ct values across studies, it is notable that participants in both inpatient and outpatient settings had relatively high Ct values (low viral loads) in their tears.

The identification of cytopathic effects in a subset of SARS-CoV-2 positive tears specimens is potentially novel. Although CPE positivity is not a direct measure of infectiousness, a prior analysis of household transmission cohorts found a link between culturable virus and SARS-CoV-2 transmissibility in nasal specimens.^10^ Our CPE findings in tears suggests that ocular fluid, including tears, could also be a potential source of SARS-CoV-2 transmission. It emphasizes the need to maintain adequate infection control precautions, particularly in situations in settings in which an individual may come in to contact with tears or ocular discharge. Although the relative importance of transmission via this mechanism is likely to be much less than via respiratory transmission, protection against contact with ocular fluid may nonetheless be an important consideration in specific settings. Current CDC guidance on SARS-CoV-2 for healthcare providers states that “HCP who enter the room of a patient with suspected or confirmed SARS-CoV-2 infection should adhere to Standard Precautions and use a NIOSH Approved particulate respirator with N95 filters or higher, gown, gloves, and eye protection (ie, goggles or a face shield that covers the front and sides of the face).”^12^ This guidance, especially the eye protection, is supported by the strong relationship we found between nasal CPE positivity and tears CPE positivity. Additionally, this relationship suggests that additional infection precautions for non-healthcare providers, such as avoiding shared linens, towels, or cosmetic products, may be warranted among individuals considered to be infectious via antigen testing or other modalities.

We observed that pre-existing histories of cancer or kidney disease were possible predictors of shedding in tears. Although these associations warrant further exploration, it is consistent that those with a weakened immune system or more severe comorbidities might be less likely to control virus in the upper respiratory tract and more likely to transmit SARS-CoV-2.^7,13^ Collectively, our data implicates a trend towards RNA-positivity in tears in patients with more severe comorbidities and factors associated with COVID-19 disease. Further investigation in cohorts with a higher burden of comorbidities may be informative.

Our study has several limitations. First, it is a convenience sample, and we therefore cannot comment on overall prevalence of SARS-CoV-2 RNA positivity in tears among the general population of people with COVID-19. Second, all samples were collected prior to the emergence of the Omicron strains, which are now predominant, and may exhibit different viral dynamics in tears. Third, we did not obtain an ophthalmologic history or history of use of ocular medications (eg, eye drops). Fourth, we did not systematically ascertain ocular symptoms in our participants, preventing us from relating the presence of RNA positivity in tears to conditions such as conjunctivitis. This is especially significant as some recent circulating strains have anecdotally been reported to be associated with ocular symptoms.^14^ Fifth, the Schirmer collection method may have a reduced sensitivity compared to a conjunctival swab.^15^ Sixth, most participants had evidence of tears RNA and CPE positivity during the infectious period; however, there was one case of postinfectious RNA positivity which may have been a contaminate or a false positive finding. Finally, the small number of tears RNA and CPE positive samples may have limited some of the ability to analyze correlations, including between nasal and tears, but also likely in terms of assessment of risk factors.

We found that tears were rarely RNA positive but, when positive, were commonly associated with culturable virus, including for up to 8 days after symptom onset. These results may have implications for infection control and prevention practices during the ongoing pandemic, particularly in settings in which ocular fluid from an individual with SARS-CoV-2 infection may be encountered.

## Supplementary Material

Supplementary Material

## Figures and Tables

**Table 1.** Demographic, clinical, and biological characteristics of participants on their day of tears RNA positivity

ID	Age range, years	Sex	Race and Ethnicity^a^	Comorbidities Reported	SARS- CoV-2 Variant	Fully Vaccinated at Time of COVID-19	Nasal PCR Result^b^	Day PSO^c^ of Tears Positive Sample	Number of COVID Symptoms	Nasal Viral RNA Load (log_10_ copies/mL)		Nasal CPE Result^b,d^	Tears Viral RNA Load (log_10_ copies/mL)		Tears CPE Result*
										N	E		N	E	
1	30-34	M	Asian	None	Pre-Delta	No	Negative	18	0	N/A^e^	N/A	N/A	2.46	3.06	Negative
2	60-64	F	White	Hypothyroidism, Melanoma, Hypertension, Asthma/COPD	Pre-Delta	No	Positive	7	14	7.99	7.10	Positive	3.15	3.06	Negative
							Positive	13	10	3.94	3.08	Negative	3.43	3.69	Negative
3	15-19	M	Hispanic/Latino	None	Pre-Delta	No	Positive	1	6	3.72	2.90	Negative	3.41	3.28	Negative
4	45-49	M	Hispanic/Latino	Kidney Disease	Pre-Delta	No	Positive	4	5	6.07	6.01	Positive	4.49	3.91	Positive
5	35-39	M	White	None	Pre-Delta	No	Positive	7	3	5.24	4.71	Positive	3.72	4.07	Positive
6	60-64	F	White	Asthma/COPD	Pre-Delta	No	Positive	4	9	7.88	6.79	Positive	3.32	3.85	Positive
7	40-44	F	Asian	None	Pre-Delta	Yes	Negative	14	2	N/A	N/A	N/A	1.98	2.45	Negative
8	40-44	F	White	Hypertension	Delta	Yes	Positive	8	1	5.96	5.93	Positive	4.77	4.35	Positive
9	25-29	M	White	None	Delta	Yes	Positive	7	10	7.73	7.05	Positive	6.69	6.66	Positive
10	35-39	F	White	Cancer, unspecified	Delta	Yes	Negative	9	0	N/A	N/A	Negative	3.87	2.98	Negative

a Race/ethnicity categories are mutually exclusive. Participants reported as American Indian or Alaska Native, Hawaiian/Pacific Islander or White were not of Hispanic/Latinx ethnicity. Participants who reported to be Asian or Black/African American may have been of Hispanic/Latinx or non-Hispanic ethnicity, but were only included for analysis under their identified race category.

b nasal sample ± 1 day of positive tears sample

c Post symptom onset (PSO)

d Cytopathic effect (CPE) assays only run on samples collected between the dE and d14 of the index case

e N/A indicates that there was no available viral load or CPE result for the nasal specimen on that day
