# Supplementary material for: Prevalence, Patterns, and Predictors of SARS-CoV-2 RNA and Culturable Virus in Tears of a Case-Ascertained Household Cohort
Source: Am J Ophthalmol. Author manuscript; Available in PMC 2025 Sep 1. (PMC11645976; doi:10.1016/j.ajo.2024.04.008)
Supplement: Supplementary Material [file NIHMS2033296-supplement-Supplementary_Material.docx]

**Supplemental Methods**

*Cytopathic effect assay (CPE)*

The methods for the CPE assay in the parent cohort have been described in detail previously.^1^ Briefly, tears specimens were used to detect culturable virus on Vero-hACE2-TMPRSS2 cells. Cells were maintained at 37 ̊C and 5% CO_2_ in Dulbecco’s Modified Eagle medium (DMEM; Gibco) supplemented with 10% fetal calf serum, 100ug/mL penicillin and streptomycin (Gibco) and 10μg/mL of puromycin (Gibco). 200uL of each specimen containing tears was added to a well of a 96-well plate and serially diluted 1:1 with DMEM supplemented with 1x penicillin/streptomycin over four additional wells. 100uL of freshly trypsinized cells, resuspended at 2.5x105 cells/mL in infection media (as above but with 2x penicillin/streptomycin, 5ug/ mL amphotericin B [Bioworld] and no puromycin), were added to each sample dilution. Cells were checked for CPE from day 2 to 5 after culture. 200uL of supernatant from each dilution series was mixed 1:1 with 2x RNA/DNA prior to RNA extraction as previously described. Presence of infectious SARS-CoV-2 was confirmed by RT-qPCR using N primers as described above without the use of an absolute standard. A Ct threshold of 3 or more cycles below that of the tears specimen that was assayed was considered to contain amplified viral RNA due to viral replication. All assays were done in the BSL3 facility at Genentech Hall, UCSF, following the study protocol that had received Biosafety Use Authorization.

*SARS-CoV-2 genome sequencing*

The methods for the SARS-CoV-2 genome sequencing in the parent cohort have been described in detail previously.^2,3^ We used the ARTIC Network amplicon-based sequencing protocol for SARS-CoV-2 and Nanopore sequencing to determine viral genome sequences. Specimens were either participant nasal specimens or the supernatants from viral cultures of tears specimens in which viral amplification has been detected by RT-qPCR. Briefly, the Arctic V3 multiplex PCR primer pools (IDT) were used to amplify viral cDNA to generate amplicons. The Native Barcode expansion kits 1–24 (Nanopore) was used label amplicons, which were then pooled and used for adaptor ligation. Libraries were run on a MinION sequencer (Oxford Nanopore Technologies) for 12–16 hours. The nCoV-2019 novel coronavirus bioinformatics protocol7 was used to determine consensus sequences and lineage determination was done using the Nexclade lineage assigner.”

**Supplemental Table 1.** Characteristics of Cohort at Baseline

| **Characteristic** | **All**  **(N = 81)** |
| --- | --- |
| **Age, median years**  **(IQR)**  **(absolute range)** | 38  (29-45)  (11-78) |
| **Female Birth Sex** | 43 (53%) |
| **Race and Ethnicity^a,b^** |  |
| White | 38 (47%) |
| Hispanic/Latino | 19 (23%) |
| Asian | 14 (17%) |
| Black/African American | 3 (4%) |
| Hawaiian/Pacific Islander | 2 (2%) |
| American Indian or Alaska Native | 2 (2%) |
| **Highest Education Completed^b^** |  |
| Any high school or less | 17 (21%) |
| Any college | 38 (47%) |
| Any graduate school | 22 (27%) |
| **Annual Household Income^b^** |  |
| $100,000 or less | 22 (27%) |
| $100,001 to $300,000 | 28 (35%) |
| More than $300,000 | 7 (9%) |
| **Body Mass Index *(kg/m^2^)*^b^** |  |
| 24.9 or less | 32 (401%) |
| 25 to 29.9 | 23 (28%) |
| 30 or greater | 22 (27%) |
| **Self-Reported Comorbid Conditions** |  |
| Any Comorbid Condition Reported | 25 (31%) |
| Autoimmune disease^b^ | 1 (1%) |
| Cancer^b,c^ | 4 (5%) |
| Diabetes^b^ | 4 (5%) |
| HIV^b^ | 0 (0%) |
| Heart attack or heart failure^b^ | 1 (1%) |
| Hypertension^b^ | 9 (11%) |
| Lung problems^b,d^ | 13 (16%) |
| Kidney disease^b^ | 2 (2%) |
| **Ever Used Tobacco^b,e^** | 11 (14%) |
| **Fully Vaccinated at Baseline^f^** | 29 (36%) |
| **Hospitalized During Acute Phase** | 2 (2%) |
| **SARS-CoV-2 Variant** |  |
| Pre-Delta | 54 (67%) |
| Delta | 27 (33%) |
| ^a^Race/ethnicity categories are mutually exclusive. Participants reported as American Indian or Alaska Native, Hawaiian/Pacific Islander or White were not of Hispanic/Latinx ethnicity. Participants who reported to be Asian or Black/African American may have been of Hispanic/Latinx or non-Hispanic ethnicity, but were only included for analysis under their identified race category.  ^b^Missing and nonresponse. Race and ethnicity: 3 missing; education: 4 missing; income: 5 missing, 19 prefer not to answer; BMI: 4 missing; any comorbidity: 1 missing; autoimmune: 1 missing; cancer: 2 missing; diabetes: 2 missing; HIV: 3 missing; heart attack: 1 missing; hypertension: 2 missing; lung problems: 1 missing; kidney disease: 3 missing; ever used tobacco: 11 missing  ^c^Cancer requiring treatment within the 2 years before COVID-19.  ^d^Asthma, COPD, emphysema, or bronchitis experienced in the 5 years before COVID-19.  ^e^Cigarettes, cigars, or any product containing tobacco in a hookah.  ^f^Participant who completed a primary series of a COVID-19 vaccine > 14 days prior to enrollment; complete primary series was defined as 2 doses for mRNA vaccines or 1 dose of Johnson&Johnson. | |

**Supplemental Table 2.** Sociodemographic and Biological Factors Associated with Tears Positivity by RT-PCR Amongst SARS-CoV-2 Infected Individuals

| **Characteristic** | **Never Tears Positive (N = 71)** | **Ever Tears Positive (N = 10)** | **Prevalence Ratio^a^** | **95% Confidence Interval^a^** | | **p-value^a^** |
| --- | --- | --- | --- | --- | --- | --- |
| **Age, median years**  **(IQR)**  **(absolute range)** | 38  (29-45)  (11-78) | 39  (32-45)  (17-61) | 1.00 | 0.96 | 1.04 | 0.937 |
| **Female Birth Sex** | 38 (54%) | 5 (50%) | 0.88 | 0.27 | 2.84 | 0.836 |
| **Race/Ethnicity** | | | | | |  |
| White | 32 (45%) | 6 (60%) | Reference | Reference | Reference | Reference |
| Hispanic/Latino | 17 (24%) | 2 (20%) | 0.67 | 0.15 | 3.02 | 0.599 |
| Asian | 12 (17%) | 2 (20%) | 0.90 | 0.20 | 4.01 | 0.895 |
| Other^b,c^ | 7 (9.9%) | 0 (0%) | N/A | N/A | N/A | N/A |
| **Education** | | | | | |  |
| Any HS or less | 15 (21%) | 2 (20%) | Reference | Reference | Reference | Reference |
| Any College | 36 (51%) | 2 (20%) | 0.45 | 0.07 | 2.95 | 0.403 |
| Any Graduate School | 17 (24%) | 5 (50%) | 1.93 | 0.42 | 8.86 | 0.397 |
| **Annual Household Income** | | | | | |  |
| $100,000 or less | 19 (27%) | 3 (30%) | Reference | Reference | Reference | Reference |
| $100,001 to $300,000 | 26 (37%) | 2 (20%) | 0.52 | 0.09 | 2.91 | 0.460 |
| More than $300,000 | 4 (6%) | 3 (30%) | 3.14 | 0.80 | 12.3 | 0.101 |
| **Body Mass Index *(kg/m^2^)*** | | | | | |  |
| 24.9 or less | 29 (41%) | 3 (30%) | Reference | Reference | Reference | Reference |
| 25 to 29.9 | 18 (25%) | 5 (50%) | 2.32 | 0.61 | 8.82 | 0.217 |
| 30 or greater | 20 (28%) | 2 (20%) | 0.97 | 0.17 | 5.39 | 0.972 |
| **Self-Reported Comorbid Conditions** | | | | | |  |
| Any Comorbid Condition Reported | 20 (28%) | 5 (50%) | 2.20 | 0.69 | 6.97 | 0.180 |
| Autoimmune^b^ | 0 (0%) | 1 (10%) | N/A | N/A | N/A | N/A |
| Cancer | 2 (3%) | 2 (20%) | 4.69 | 1.43 | 15.35 | 0.011 |
| Diabetes^b^ | 4 (6%) | 0 (0%) | N/A | N/A | N/A | N/A |
| HIV^b^ | 0 (0%) | 0 (0%) | N/A | N/A | N/A | N/A |
| Heart attack or heart failure^b^ | 1 (1%) | 0 (0%) | N/A | N/A | N/A | N/A |
| Hypertension | 7 (10%) | 2 (20%) | 1.94 | 0.48 | 7.84 | 0.350 |
| Lung problems | 11 (15%) | 2 (20%) | 1.29 | 0.31 | 5.44 | 0.730 |
| Kidney disease^b^ | 1 (1%) | 1 (10%) | 4.75 | 1.02 | 22.2 | 0.048 |
| **Ever Used Tobacco**^b^ | 11 (15%) | 0 (0%) | N/A | N/A | N/A | N/A |
| **Fully Vaccinated at Baseline** | 25 (35%) | 4 (40%) | 1.20 | 0.36 | 3.92 | 0.768 |
| **Hospitalized During Acute Phase**^b^ | 2 (3%) | 0 (0%) | N/A | N/A | N/A | N/A |
| **SARS-CoV-2 Variant** | | | | | |  |
| Pre-Delta | 47 (66%) | 7 (70%) | Reference | Reference | Reference | Reference |
| Delta | 24 (34%) | 3 (30%) | 0.86 | 0.24 | 3.08 | 0.813 |
| **Nasal Viral Load (Nucleocapsid)^d^** | N/A | N/A | 1.02 | 0.80 | 1.30 | 0.856 |
| ^a^Prevalence Ratios (PRs), 95% Confidence Intervals (CIs), and p-values were obtained using modified Poisson GLM regression models  ^b^N/A = Not Applicable, assigned to variables where there were insufficient numbers to estimate prevalence ratios  ^c^Other race category encompasses the following racial categories: Black/African American, Hawaiian/Pacific Islander, and American Indian or Alaska Native  ^d^Adjusted for both the age of participant and day post-symptom onset | | | | | | |

**Supplemental Figure 1.** Flow diagram of participant inclusion.


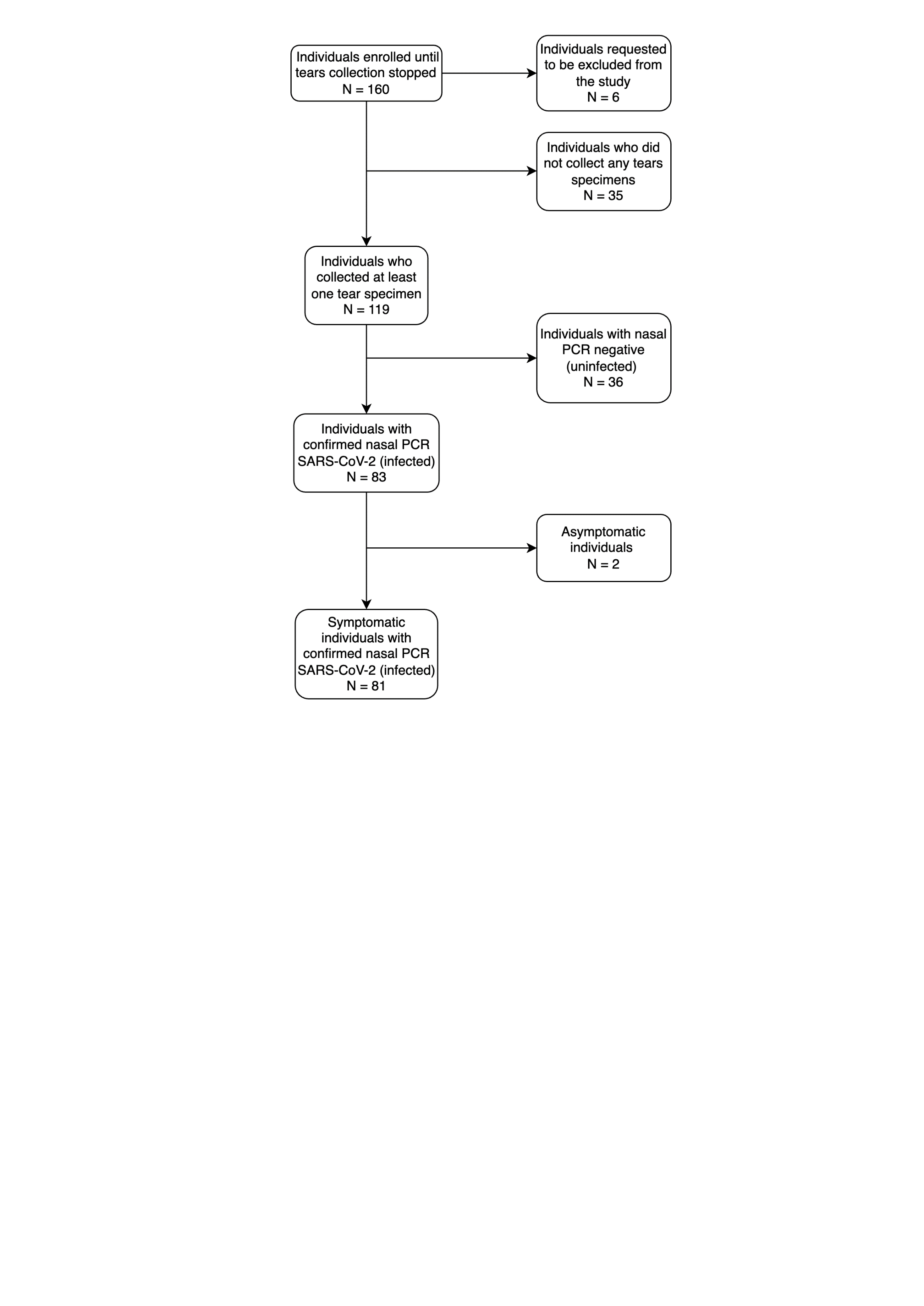


**References**

1. Garcia-Knight M, Anglin K, Tassetto M, et al. Infectious viral shedding of SARS-CoV-2 Delta following vaccination: A longitudinal cohort study. *PLoS Pathog*. 2022;18(9):e1010802. doi:10.1371/journal.ppat.1010802

2. Tyson JR, James P, Stoddart D, et al. Improvements to the ARTIC multiplex PCR method for SARS-CoV-2 genome sequencing using nanopore. Published online September 4, 2020:2020.09.04.283077. doi:10.1101/2020.09.04.283077

3. Aksamentov I, Roemer C, Hodcroft EB, Neher RA. Nextclade: clade assignment, mutation calling and quality control for viral genomes. *J Open Source Softw*. 2021;6(67):3773. doi:10.21105/joss.03773
